# Experimental Study on the Effect of Humidity on the Mechanical Properties of 3D-Printed Mechanical Metamaterials

**DOI:** 10.3390/polym17212938

**Published:** 2025-11-03

**Authors:** Qian Sun, Xiaojun Tan, Jianhao Man, Shuai Li, Zeeshan Ali, Kaiyang Yin, Bo Cao, Christoph Eberl

**Affiliations:** 1 School of Civil Aviation, Northwestern Polytechnical University, Xi’an 710072, China; sunqian@nwpu.edu.cn (Q.S.); xiaojun_tan1@163.com (X.T.); zeeshanali390@yahoo.com (Z.A.); 2 Tianjin Key Laboratory of Power Transmission and Safety Technology for New Energy Vehicles, School of Mechanical Engineering, Hebei University of Technology, Tianjin 300130, China; manjianhao703@163.com; 3 Department of Engineering Mechanics, Faculty of Civil Engineering and Mechanics, Kunming University of Science and Technology, Kunming 650500, China; lishuai@kust.edu.cn; 4 State Key Laboratory of Advanced Marine Materials, Ningbo Institute of Materials Technology and Engineering, Chinese Academy of Sciences, Ningbo 315201, China; 5 Cluster of Excellence livMatS @ FIT—Freiburg Center for Interactive Materials and Bioinspired Technologies, University of Freiburg, Georges-Köhler-Allee 105, 79110 Freiburg, Germany; chris.eberl@iwm.fraunhofer.de; 6 Laboratory for Micro and Materials Mechanics, Department of Microsystems Engineering-IMTEK, University of Freiburg, 79110 Freiburg, Germany; 7 Fraunhofer Institute for Mechanics of Materials (IWM), 79108 Freiburg, Germany

**Keywords:** mechanical metamaterial, humidity effects, 3D-printed polymers, mechanical performance, fused filament fabrication (FFF)

## Abstract

In this study, six common fused filament fabrication (FFF) polymers—PEEK, PLA, PETG, ABS, Nylon, and TPU—were acclimatized at 15%, 45%, and 95% relative humidity (RH) to characterize tensile behavior, including Young’s modulus, maximum strain, and ultimate tensile strength. Separately, mechanical metamaterial samples at relative densities (RD) of 25%, 35%, and 45% were tested in compression at the same RH levels to evaluate stiffness, strength, and Poisson’s ratio. The water absorption process can generally be divided into different stages—rapid uptake (0–12 h), a plateau (12–60 h), and a late rebound (60–100 h)—with a total uptake ranking of Nylon > PETG > PLA ≈ ABS > TPU ≈ PEEK. Samples under tensile and compressive tests show a great difference between properties at different RD and RH levels. Poisson’s ratio indicates that material responses remain predictable at low-to-moderate RH, whereas high RH serves as a critical threshold inducing abrupt Poisson’s ratio behavioral shifts. This study provides systematic validation for the application of 3D-printed metamaterials under varying humidity conditions, such as biomedical implants in human body.

## 1. Introduction

Mechanical metamaterials leverage precisely engineered geometries and internal porosity to achieve exceptional mechanical performance—combining low density, high strength, and tunable deformation [1,2]. With the maturation of fused filament fabrication (FFF) and other additive manufacturing techniques [3,4], a variety of polymer filaments are now widely employed to fabricate functional metamaterial components. Unlike conventional uniform-density structures, metamaterial architectures can maintain load-bearing stiffness while, through careful pore distribution and unit-cell design, delivering complex behaviors such as energy absorption [5], negative Poisson’s ratio [6], and mechanical anisotropy [7]. These capabilities have demonstrated immense potential in aerospace [8], wearable devices [9], and robotics [10]. Negative Poisson’s ratio metamaterials—commonly known as “auxetics”, following the seminal work of Evans et al. [11,12]—form a unique subclass of mechanical metamaterials whose unusual microstructures impart a suite of exceptional properties [13,14]. By virtue of re-entrant hinges, rotating units, or chiral motifs, auxetics demonstrate pronounced indentation resistance [15], remarkable shear [16] and fracture resistance [17], and outstanding energy-absorption capacity [18]. Geometrically, these architectures fall into several major categories—foam-derived structures [19], re-entrant honeycombs [20], and chiral and anti-chiral lattices [21]—each offering tailored mechanical responses. Such versatility has enabled their integration into a broad array of devices and systems, including cushioning pads [22], intelligent filters [23], and sensing platforms [24].

**Figure 1:**
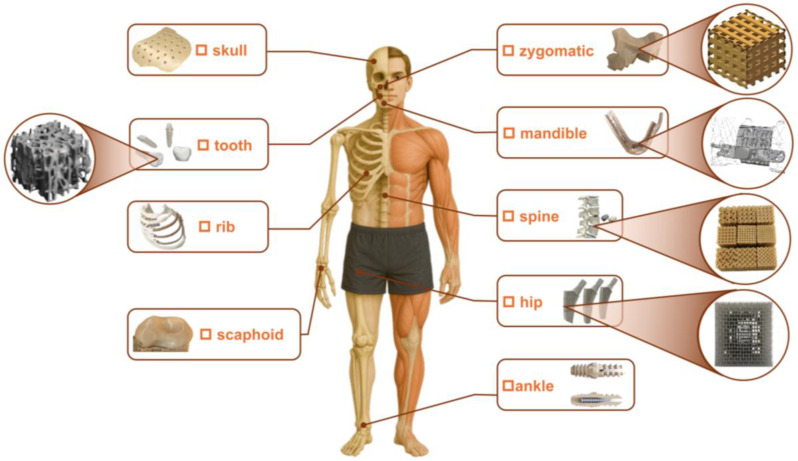
Current and prospective applications of 3D-printed metamaterials within the human body, including skull [45], tooth [46], rib [46], scaphoid [45], zygomatic [45], mandible [47], spine [46], hip [26], ankle [46], etc.

Except for those mentioned above, many metamaterial components must operate under variable humidity conditions [25] in practical applications. For instance, implants in contact with biological tissues [26,27,28] encounter nearly 100% body-fluid environments, marine structures [29] must withstand high-salt fog humidity, and industrial or domestic uses [30] often face seasonal or localized moisture fluctuations. Some examples of current and prospective applications of 3D-printed metamaterials within the human body can be found in Figure 1. It should also be noted that tissue fluids in the human body cannot be simply represented by different humidity levels. Therefore, to investigate biomedical applications, it is essential to control the environment using actual physiological fluids, such as blood. Humidity governs polymer water uptake and distribution, which can in turn alter key mechanical properties. Existing studies have largely focused on the effect of filament moisture content on printing quality or on the water absorption behavior of finished parts. Wang et al. [31] showed that varying filament moisture levels influence the surface quality of FFF-printed Polyether Ether Ketone (PEEK), and that increased moisture in the filament reduces tensile strength, density, and hardness. Kariz et al. [32] found that wood–polylactic acid (PLA) filaments with higher wood content absorb more water, exhibit greater dimensional swelling, and display lower elastic modulus when conditioned at 33%, 65%, and 87% relative humidity (RH). Leite et al. [33] examined the influence of a sealing protective treatment on the water absorption behavior and mechanical properties of acrylonitrile butadiene styrene (ABS) components manufactured using FFF. Their results demonstrated that an acrylic-based coating effectively reduced porosity while maintaining the mechanical integrity of the specimens. Moreno Nieto et al. [34] found that for 3D-printed polyethylene terephthalate glycol (PETG) and PLA, the water absorption process reaches equilibrium within a few weeks when exposed to saturated aqueous solutions containing marine salts and sugars. Banjo et al. [35] investigated the effects of humidity and temperature on 3D-printed Nylon, carbon fiber-reinforced Nylon, and PLA. They found that Nylon-based materials absorb over ten times more water than PLA, with moisture uptake directly linked to reduced flexural properties—Nylon’s flexural modulus decreased by up to 60% after 7 days of immersion. PLA showed minimal property changes at 21 °C but experienced significant physical degradation after 7 days at 70 °C. Xu et al. [36] studied the effect of water on thermoplastic polyurethane (TPU) blends with varying hard-to-soft segment ratios. They found that water immersion softens TPU due to plasticization, hydrogen bond disruption, and new water-induced crosslinking. After partial water loss, the modulus increases compared to the dry state, indicating a hardening effect. Mian et al. [37] evaluated water absorption of 3D-printed ABS, PLA, TPU, PETG, and polypropylene (PP). All tested polymers showed strong hydrophobicity with minimal water absorption even after 192 h of immersion, with PP absorbing the least and PETG the most, confirming their good suitability for knee orthoses. Reuvers et al. [38] found that the nuclear magnetic resonance signal of a 200 µm thick Nylon-6 film exhibited a highly nonlinear relationship with its water content. Pai et al. [39] found that with increasing molding and annealing temperatures, the hygroscopicity of Nylon-6,6 decreases, while increased moisture absorption due to plasticization reduces strength and stiffness but enhances ductility and impact resistance. Boubakri et al. [40] found that TPU samples immersed in water for up to six months at a set temperature of 70 °C exhibited reductions in both elastic modulus and stress. However, most of these studies did not link humidity with mechanical properties, especially in the case of 3D-printed mechanical metamaterials, as the effect of moisture on the mechanical behavior of various 3D-printed mechanical metamaterials remains underexplored.

Mechanical metamaterials are inherently sensitive to environmental conditions due to their precisely engineered internal architectures [41]. Unlike conventional bulk materials, these structures rely on periodic or cellular designs [42] in which voids, interfacial bonding, and deformation pathways are highly susceptible to moisture. This sensitivity varies markedly among polymer types, as their water uptake, retention, and response depend on material chemistry, porosity, and processing. At present, a considerable amount of research has been conducted on PEEK; for example, biomedical implants [43,44] based on PEEK are exposed to near-saturated humidity in vivo, where prolonged contact with body fluids can induce water absorption, dimensional swelling, and network softening. PEEK and carbon fiber-reinforced PEEK both have stiffness close to cortical bone, reducing stress shielding and aiding osseointegration. Their radiolucency and absence of artifacts in X-ray, computed tomography (CT), and magnetic resonance imaging (MRI) enable clear monitoring of bone healing. In addition, PEEK [43] offers excellent biocompatibility, wear resistance, fatigue resistance, corrosion resistance, and machinability, and is lighter than metallic materials. These advantages have led to widespread use in trauma, spinal, and joint applications. Understanding each material’s mechanical behavior under various humidity conditions is critical for ensuring implant safety and long-term function, since humidity-driven softening can lead to excessive deformation or stiffness mismatch and thereby destabilize the device or concentrate stress at tissue interfaces [25]. Additionally, the interplay among specific surface area, structural architecture, and absorption also influences structural strength, dimensional stability, and load-bearing capacity and must be accounted for in design. Not only for PEEK, but also for other materials, such as ABS, TPU, Nylon, PLA, and PETG, a considerable number of studies exist; however, the testing conditions are not consistent, making direct comparisons difficult. Therefore, a comprehensive understanding of the interplay between material selection and ambient humidity is essential for the durable, accurate, and reliable performance of mechanical metamaterials across diverse applications.

In this work, the performance of polymer-based metamaterials will be systematically evaluated as a function of RH and RD—two key factors governing moisture uptake, interlayer bonding, and the overall mechanical response, which have not been comprehensively investigated before. This study aims to systematically quantify humidity-driven changes in mechanical response of architected lattices by measuring water-uptake kinetics under immersion to determine equilibrium moisture content and diffusivity for each polymer, characterizing tensile properties as a function of controlled RH to isolate hygroscopic effects on modulus, strength, and ductility, and evaluating compressive performance of lattices across a combination of RD and RH while recording full-field strain and Poisson’s ratio via digital image correlation (DIC). Based on the above research, it is speculated that the negative Poisson’s ratio of each material varies with changes in RD and RH. However, whether this relationship is positive or negative still requires systematic investigation.

To systematically investigate the effect of humidity on the mechanical performance of 3D-printed mechanical metamaterials, this study selected six common FFF polymers, namely ABS, TPU, Nylon, PLA, PEEK, and PETG, and conducted water absorption tests to quantify their moisture uptake behavior. Since the present study is not aimed at biomedical exploration, physiological fluids to simulate environmental changes were not employed; instead, the more easily controlled parameter of RH to examine the environmental dependence of the materials was used. Then, humidity-dependent tensile tests were carried out at 15%, 45%, and 95% RH to assess the material’s mechanical performance. Compression tests at 15%, 45%, and 95% RH for mechanical metamaterials were performed on specimens with relative densities (RDs) of 25%, 35%, and 45%. Our work elucidates how RH and RD influence metamaterial properties and provides practical guidance on material selection and geometric optimization for high-humidity applications. 

## 2. Experimental Procedures

### 2.1. Specimen Preparation

The suppliers of ABS, TPU, Nylon, PLA, PEEK, and PETG used in this study are listed in Table 1. Filaments of different colors may affect performance differently [48]; however, this study focuses on the base material itself. Prior to printing, all filaments were dried in a PrintDry Filament Dryer PRO 3 (PrintDry Co, Ltd., Windsor, ON, Canada) to eliminate moisture and ensure optimal print quality. PEEK specimens were printed on a CREATBOT F160 printer (Henan Creatbot Co, Ltd., Zhengzhou, China). Because PEEK is prone to warping during FFF [49], a Magig 3D adhesive (glue stick) was applied to the build plate to improve bed adhesion. All other materials were printed on a Prusa i3 MK3S+ at room temperature. Printing parameters are also given in Table 1.

**Figure 2:**
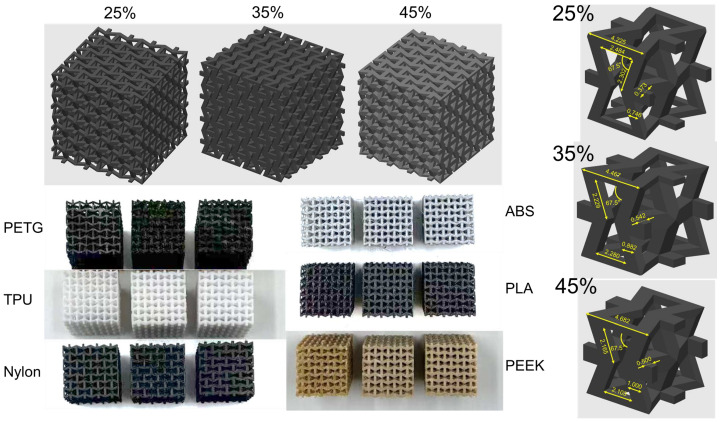
Sample structures used for compression testing of 3D-printed mechanical metamaterials. The upper side shows the 3D-printing models, from left to right, at 25%, 35%, and 45% RD. The lower side shows the actual 3D-printed samples at 25%, 35%, and 45% RD, respectively, from left to right. The right side shows the unit-cell dimensions for lattices at 25%, 35%, and 45% RD.

Solid tensile specimens were fabricated according to ASTM D638 [50] Type I dimensions (overall length of 165 mm, gauge length of 50 mm, width of 13 mm, and thickness of 3.2 mm). The tensile specimen is flat in printer’s X-axis. The infill pattern was a 45° grid along the X-direction. The nozzle size is 0.4 mm. The layer height is 0.3 mm. The perimeter count is set as 1. Compressive specimens measured 32 × 32 × 32 mm and were produced at RDs of 25%, 35%, and 45%, respectively. The specific structure [51] can be found in Figure 2.

### 2.2. Measurements

All moisture measurements were conducted strictly in accordance with the ASTM D570 standard [52]. For every absorption test, flat solid specimens with a size of 60 × 60 × 1 mm were fully submerged in water for the specified duration and then removed from the water one at a time. Then, all surface water was wiped off with a dry cloth, and the specimens were weighed to the nearest 0.001 g immediately. To ensure accuracy, each test was repeated three times. At regular intervals, each sample was weighed on a KERN ABT 120-5DNM (KERN & SOHN GmbH, Balingen, Germany) balance to monitor moisture uptake.

Dynamic Mechanical Analysis (DMA) was conducted once on the original solid specimens without humidity conditioning, with the dimension of 30 × 5 × 1 mm by DMA8000 (PerkinElmer, Waltham, MA, USA), to characterize their inherent viscoelastic properties and establish a dry/ambient baseline for subsequent humidity studies. The tests were conducted with a heating rate of 2 °C/min and a frequency of 1 Hz. A low heating rate reduces thermal gradients and phase-lag between sample and furnace, and it sharpens viscoelastic transition peaks (improving the accuracy of indicated glass-transition temperatures). Single cantilever bending was applied during the analysis. The temperature range for the tests spanned from −50 °C to 250 °C.

To evaluate the mechanical performance of various 3D-printed polymer materials and to assess the behavior of negative Poisson’s ratio metamaterials fabricated from different materials, samples were conditioned at 45% and 95% RH for 24 h using a programmable HCP50 chamber (Memmert GmbH, Schwabach, Germany) and monitored using Thermo-/Hygrometer (Renkforce, Hirschau, Germany). Additionally, a separate batch of samples were also dried using a PrintDry and monitored using Thermo-/Hygrometer (Renkforce), with humidity controlled at 15% to serve as the dry control group. Therefore, three groups of samples conditioned at 15%, 45%, and 95% RH were prepared, respectively. Samples were periodically weighed using an analytical balance until the change in mass between consecutive 24 h measurements became negligible, indicating that the specimen had reached its equilibrium moisture content. After taking specimens from the chamber, the mechanical tests were conducted immediately. Although RH may fluctuate slightly during this process, the change is negligible because the transfer time is short. In future work, the environmental chamber will be mounted on the universal testing machine to achieve more precise RH control. Tensile and compression tests were carried out on an universal testing machine (ZwickRoell Pte. Ltd, Singapore). For PEEK, PLA, PETG, and ABS, the tensile rate was set to 0.05 mm/s; for Nylon and TPU, a higher rate of 0.5 mm/s was used to accommodate their large elongation at break. Compressive tests were performed at 0.03 mm/s. Prior to testing, all specimens were speckle-coated with edding 5200 permanent black-and-white spray for DIC, and deformation was recorded using a 4K camera (Blackmagic Design, Fremont, CA, USA) with a 50 mm macro lens and analyzed by GOM Correlate software (version 2019). The subset size is set as 25 pixels, and the step is set as 1 pixel. The gauge region for analysis was defined as the central 80% of the gauge length (axial direction) and the central 80% of the specimen width. Then, transverse and longitudinal strains of the samples during deformation can be measured precisely to facilitate the calculation of Poisson’s ratio (here, the averaged value was adopted), and they also provide qualitative visual evidence of local instabilities and slip deformation. Both tensile and compressive tests were repeated at least three times.

## 3. Results and Discussions

### 3.1. Preliminary Results of 3D-Printed Mechanical Metamaterials for Compressive Tests

**Figure 3:**
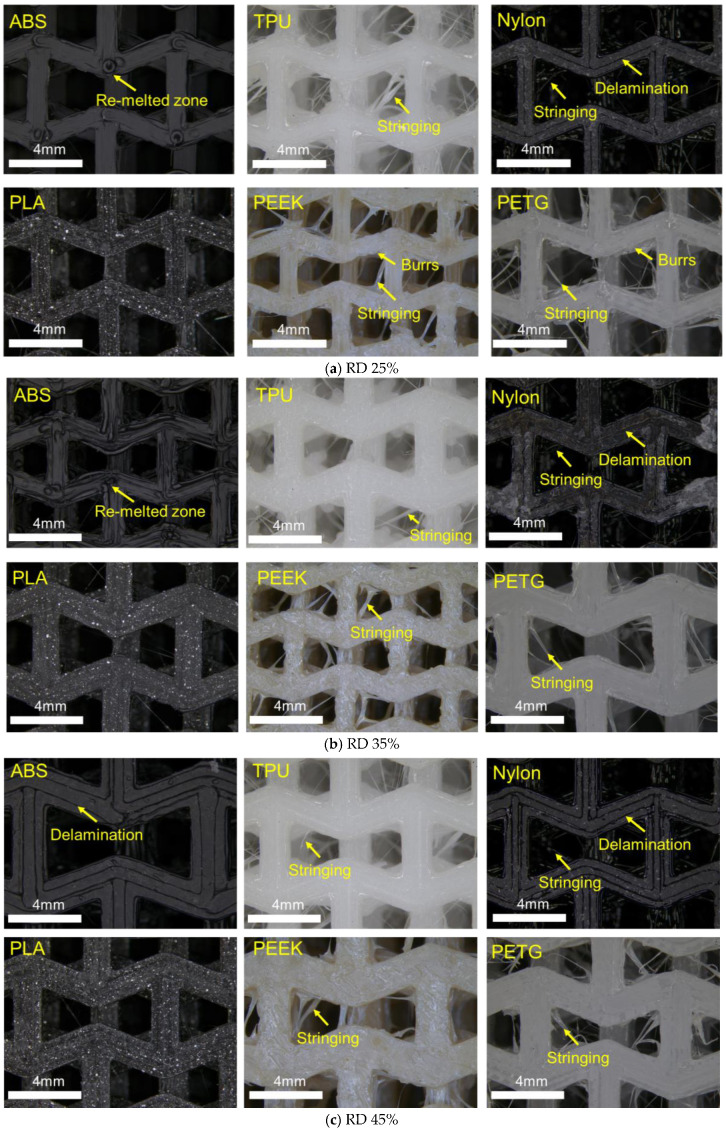
Enlarged graphs of the compression specimens observed from the top at RDs of (**a**) 25%, (**b**) 35%, and (**c**) 45%.

Enlarged graphs of the 3D-printed samples at different RDs (25%, 35%, and 45%) are shown in Figure 3. At 25% and 35% RD, the ABS sample exhibits a pronounced re-melted zone due to the nozzle passage for the realization of the layer, while at 45% RD, these zones disappear, and delamination is observed. The visible delamination arises because the print head did not extrude sufficient material to fully fill the framework of the individual section in a single pass (i.e., under-extrusion). Such under-extrusion can result from the nominal extrusion settings (flow/extrusion multiplier, print speed, and temperature) and from the intrinsic rheological properties of the filament (melt viscosity, fillers/additives, and feed behavior). Nylon samples show persistent delamination across all RDs. TPU, PEEK, and PETG samples display extensive stringing with burr formation. As RD increases, both the types and severity of defects exhibit slight differences. These defects may compromise the material’s mechanical performance.

**Figure 4:**
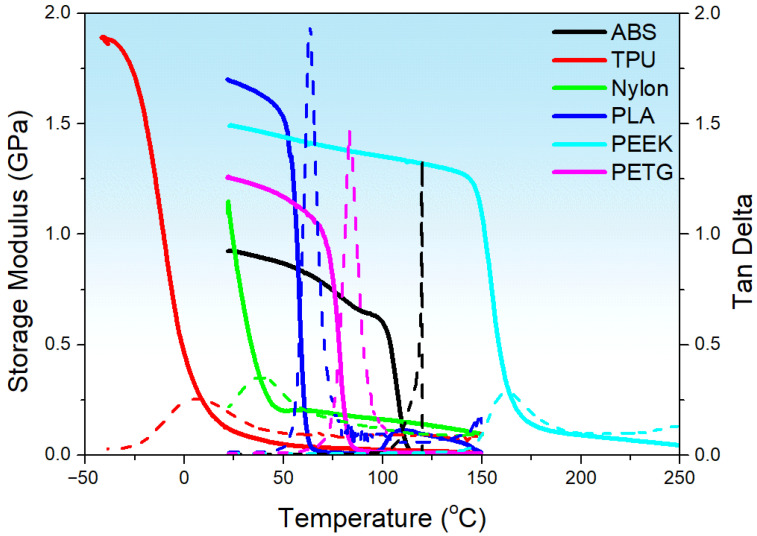
The storage modulus and tan delta of various samples under single cantilever bending. The solid line corresponds to the storage modulus, and the dashed line corresponds to tan delta.

Figure 4 shows the storage modulus and tan delta of various samples under single-cantilever bending. The DMA data presented here represent the dry/ambient baseline viscoelastic response of the polymers and are used only to provide a baseline mechanical characterization. Direct correlation of humidity-driven mechanical behavior with DMA metrics requires DMA to be performed under the same humidity conditioning and will be conducted in future work. The results indicate glass transition temperatures of TPU (~−10 °C), Nylon (~50 °C), PLA (~60 °C), PETG (~80 °C), ABS (~105 °C), and PEEK (~145 °C). At ambient temperature, PEEK exhibits the highest storage modulus (~1.5 GPa), followed by PLA and PETG (~1.3 GPa and ~1.1 GPa, respectively). Nylon and ABS show intermediate moduli (~0.8–1.0 GPa), whereas TPU has the lowest (<0.2 GPa). The damping factor (tan δ) peaks highest for PLA (>1.8), indicating strong energy dissipation, with PETG and ABS at moderate levels (~1.3–1.4), as well as Nylon, PEEK, and TPU at lower levels (~0.2–0.4). These observations suggest that PEEK is suited for high-temperature, high-stiffness applications; ABS and PETG are appropriate up to ~80–100 °C; PLA and Nylon are best used below 60 °C; and TPU is optimal for low-temperature or high-damping applications.

### 3.2. Water Absorption Testing of the Materials

The increase in weight percent, $\eta_{w}$, can be calculated by the following equation:

(1) $$\eta_{w}=\frac{g_{w}-g_{r}}{g_{r}}\times 100$$

where $g_{w}$ and $g_{r}$ represent wet weight and dry weight, respectively.

**Figure 5:**
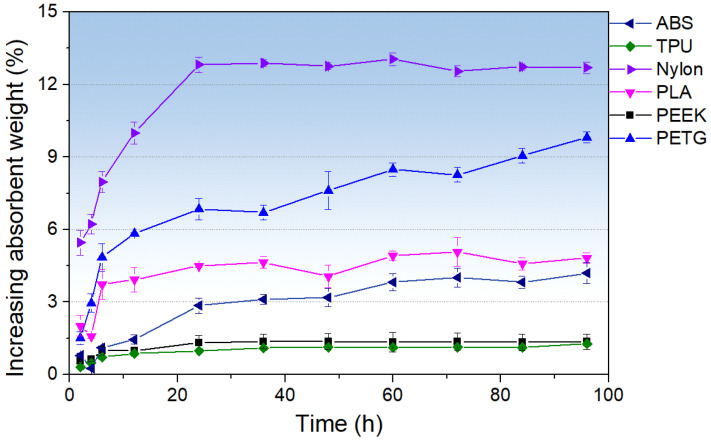
Temporal evolution of specimen weight during water absorption for ABS, TPU, Nylon, PLA, PEEK, and PETG. The error bars denote the standard error (SE) of the mean absorbent performance.

Figure 5 shows the temporal evolution of specimen weight during water absorption for ABS, TPU, Nylon, PLA, PEEK, and PETG. It should be noted that all materials used in the water absorption tests were 3D-printed samples. In the first 0–12 h, all samples undergo rapid mass gain driven by surface capillarity and the filling of large pores, with Nylon exhibiting the most pronounced increase (≈10% at 12 h); a moderate uptake in PETG, PLA, and ABS (rising to ≈1.5%~5.8% by 12 h); and minimal absorption in TPU and PEEK (remaining near ≈0.9%). Between 12 and 60 h, weight fluctuations reflect alternating cycles of surface moisture evaporation and re-adsorption; however, an overall upward trend is evident. Beyond 60 h, deeper voids continue to fill while residual surface water is recaptured, producing a slight rebound: Nylon stabilizes at ≈12.7%; PETG, PLA, and ABS increase to ≈9.3%, 9.8%, 4.8%, and 4.2%, respectively; and TPU and PEEK rise only to ≈1.3%. Compared to the water absorption properties reported by material suppliers [53] for non-3D-printed materials—only ABS, PEEK, and PETG—the 3D-printed materials exhibit markedly increased water uptake, especially PETG. Although non-3D-printed PETG absorbs less water than ABS and PEEK, the 3D-printed PETG shows far greater water absorption capacity than both other polymers. In general, a thickness-normalized diffusion analysis [54] and a Fickian fit per material are required to evaluate whether a material is more prone to water absorption. However, in this study, the water absorption test represents only a minor aspect of the work. The main focus is on the effects of different RH and RD conditions on the mechanical properties of the mechanical metamaterials. Therefore, only mass measurements and a calculation of $\eta_{w}$ were conducted to quickly assess their water absorption behavior. A more systematic and detailed study on water absorption will be carried out in future work.

### 3.3. Effect of Relative Humidity on the Tensile Properties of Materials

**Figure 6:**
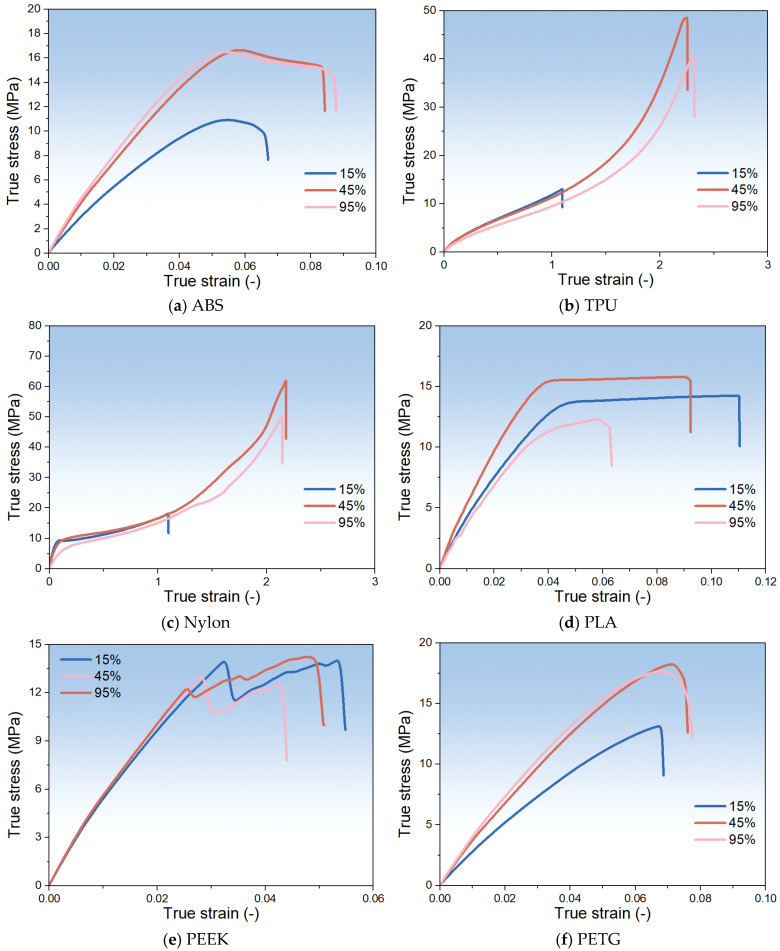
The true stress–true strain curves of various materials obtained under tensile loading at 15%, 45%, and 95% RH.

Figure 6 presents the true stress–true strain curves obtained under tensile loading at 15%, 45%, and 95% RH. Across all materials, the tensile response follows a mid-humidity optimum pattern, which reflects the role of moisture distribution within micro-voids and between printed layers. In Nylon (Figure 6c) and TPU (Figure 6b), the greatest displacement and highest tensile strength occur at 45% RH, with clear gains over both lower and higher humidity levels. This result indicates that TPU is a highly humidity-sensitive material, despite its poor water absorption performance (Figure 5). PETG and ABS exhibit increased Young’s modulus and tensile strength at 45% and 95% RH compared to 15% RH, though differences among these humidity conditions are modest. PLA shows a slight performance fluctuation at 45% RH but lower displacement and load than the dry and high-humidity states, while PEEK likewise shows no improvement at moderate humidity. These observations can be attributed to partial capillary condensation and/or matrix absorption, leading to effective pore occlusion at moderate humidity [55], which reduces local stress concentrations [56] and forms a thin lubricating film at layer interfaces to enhance interlayer adhesion and facilitate internal slip, particularly in high-absorption materials such as Nylon and TPU; excessive moisture at higher humidity, however, may generate pores [56,57] or local stress-bearing defects [56] that flatten or slightly reduce gains in stiffness and strength. Doyle and Weidlich [58] have pointed out that pore filling is associated only with liquid water and not with humidity. However, pore filling can also result from capillary condensation or from local swelling of the polymer matrix, reducing the effective cross-sectional area. These two mechanisms produce mechanical effects similar to pore filling. Materials with inherently low porosity or limited water uptake (e.g., PEEK) show minimal sensitivity to ambient humidity. In addition, it is clear that the true stress–strain curves show a great difference in TPU and Nylon; as evidenced, the repeated results are shown in Appendix A.1.

**Figure 7:**
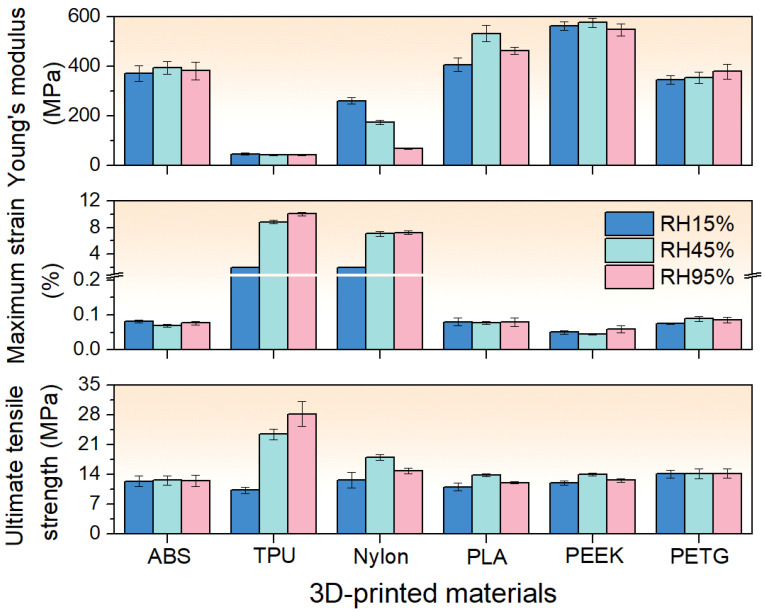
The Young’s modulus, maximum strain, and ultimate tensile strength (UTS) of each material at 15%, 45%, and 95% RH. The error bars denote the SE of the mean mechanical properties.

Figure 7 summarizes the Young’s modulus, maximum strain, and ultimate tensile strength (UTS) of each material at 15%, 45%, and 95 % RH. The detailed value can be found in Appendix A.2. From the figure, it can be seen that ABS and PEEK samples maintain a nearly constant Young’s modulus, maximum strain, and UTS across RH levels. This indicates that ABS and PEEK exhibit excellent stability under varying humidity conditions. Young’s modulus of TPU remains stable, whereas both its maximum strain and UTS increase with RH, making it the most humidity-sensitive material. Nylon also exhibits significant sensitivity to humidity: its Young’s modulus decreases as humidity rises, its maximum strain increases, and its UTS peaks at 45% before declining at 95% RH. PLA at 45% RH shows a slightly higher Young’s modulus and UTS, although the increase is marginal. PETG also shows a relatively stable performance, except for a slight increase in Young’s modulus with RH.

### 3.4. Effect of Relative Density and Relative Humidity on the Mechanical Behavior of the Mechanical Metamaterials

Because the metamaterial’s filling density is below 100%, significant internal voids are present. Therefore, true stress calculations must incorporate relative density, as shown below.

(2) $$\sigma_{E}=\frac{F}{A}=\frac{F}{l\times w\times RD}\;,\;\sigma_{t}=\sigma_{E}\left(\varepsilon_{t}+1\right)$$

where $\sigma_{E}$ is the engineering stress; F is the force; A is the cross-sectional area; l and w are the length and width of sample; RD is replaced with the value of 25%, 35%, and 45%; $\sigma_{t}$ is the true stress; and $\varepsilon_{t}$ is the true strain.

**Figure 8:**
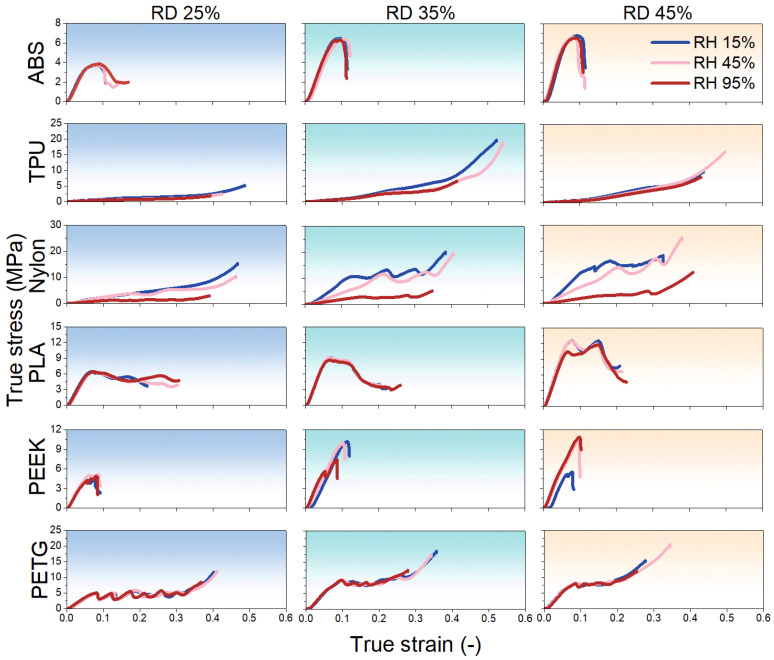
The representative compressive true stress–true strain curves at 15%, 45%, and 95% RH for all materials across three RDs (25%, 35%, and 45%). From left to right, the results at 25%, 35%, and 45% RD are presented. From top to bottom, the results correspond to ABS, TPU, Nylon, PLA, PEEK, and PETG, respectively.

Figure 8 presents the representative compressive true stress–true strain curves at 15%, 45%, and 95% RH for all materials across three RD (25%, 35%, and 45%). Since the results show good reproducibility, only representative results have been selected. Let us first examine how RH affects the mechanical performance of these metamaterials. For ABS, samples at different RDs exhibit nearly identical mechanical responses across all RH levels, indicating excellent humidity–performance stability. Compared with tensile properties (Figure 6), the same stable tendency is obtained. For TPU, increasing RH reduces peak stress, especially at 95% RH, at different RDs. The decreasing tendency of peak stress with RH is opposite to that under tension (Figure 6). Nylon follows a similar trend, with stress levels declining significantly as RH increases. It is also clear that Young’s modulus decreases with increasing RH, which is similar to that under tension (Figure 6). PLA samples at all RDs display no obvious change in mechanical behavior with RH, demonstrating very high humidity–performance stability, the same as under tension, suggesting great stability at different loading conditions. PEEK samples at 25% RD remain essentially unchanged with RH. At 35% RD, peak stress is lowest at 95% RH, whereas at 45% RD, peak stress is lowest at 15% RH. This irregular variation, which lacks a clear pattern, is quite different from tension and may be due to the influence of the structure. PETG samples at different RDs maintain similar mechanical performance across all RH levels, the same as tension, also showing an excellent stability. In all cases, maximum strain shows no significant variation with RH when compared to peak stress.

**Figure 9:**
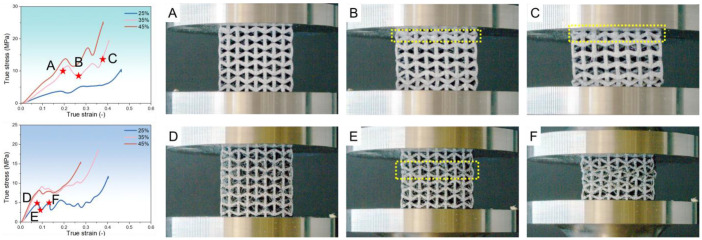
(**A**–**C**) Compression–deformation images of a Nylon sample with 35% RD under RH 45% during the deformation process. (**D**–**F**) Compression–deformation images of a PETG sample with 25% RD under RH 15% during the deformation process.

The effect of RD on the mechanical behavior of each metamaterial is now examined (Figure 8). For ABS, at all RH levels, samples with 25% RD exhibit a lower peak stress. Samples at 35% and 45% RD show nearly identical stress–strain curves. For TPU, the 25% RD samples also have a reduced peak stress. There is no clear trend in their maximum strain. For Nylon, 25% RD samples display a steady increase in true stress as true strain grows. However, both 25% and 35% RD samples show fluctuations in their true stress–strain curves. For example, at 45% RH and 35% RD, points A, B, and C in Figure 9 mark key stages. In the first stage, stress rises with strain under uniform compression. As stress increases further, the unit cell bends outward (yellow dashed region in Figure 9B), losing contact with adjacent cells and causing a temporary drop in true stress. With continued compression, that cell contacts the upper platen, resists deformation, and true stress climbs sharply again. For PLA, peak stress increases with RD at every RH level. This suggests that PLA metamaterials are highly sensitive to RD changes. A previous study [59] has also demonstrated that brittle lattice metamaterials (rigid 10k resin) with a Kelvin lattice structure exhibit significant variations in stress and fracture strength with changes in RD. For PEEK, larger RD samples generally show higher peak stress, but no consistent pattern emerges. Notably, at 15% RH, the 35% RD sample records the highest peak stress. For PETG, 25% RD samples exhibit lower stress and the same fluctuations seen in Nylon. For example, at 15% RH and 25% RD (points D, E, and F in Figure 9), unit cells compact and draw closer under compression, and then a unit cell (yellow dashed region in Figure 9E) slips and bends outward, causing a brief stress drop. As the structure restabilizes, stress rises again. Further compression leads to unit cells overlapping, which eliminates fluctuations and yields a steadily rising stress curve. In summary, all metamaterials show the lowest peak stress at 25% RD. Increasing RD raises peak stress across the board. For ABS, TPU, and PETG, the mechanical behavior at 35% and 45% RD is similar. In contrast, Nylon, PLA, and PEEK exhibit a stronger dependence of mechanical performance on RD—especially PLA, where the differences are most pronounced.

RD is a key structural parameter determining the mechanical strength of 3D-printed metamaterials. As RD increases from 25% to 35% and 45%, the cell wall thickness or strut cross-sectional area grows significantly. This enhances the overall stiffness and load-bearing area, leading to higher peak stress. At a low RD (25%), thin-walled structures are prone to local bending, buckling, or instability. Minor defects from the manufacturing process can concentrate internal stress, causing a sharp strength reduction. At a high RD, increased cell contact surfaces and load paths help disperse stress concentration and reduce local instability. As a result, materials such as ABS, TPU, and PETG show highly similar stress–strain curves at 35% and 45% RD. In contrast, PLA, PEEK, and Nylon, which are more sensitive to structural geometry [60], exhibit more noticeable differences in mechanical performance at different RD levels.

Furthermore, the metamaterials’ performance differences result from the combined effects of structural geometry and RH. ABS and PLA are structure-dominated metamaterials, with performance nearly entirely determined by RD. Therefore, it may be advisable to modify the metastructure of ABS and PLA to reduce their sensitivity to various RH in environments. Nylon is a humidity-sensitive metamaterial, with strength significantly influenced by RH. TPU, PEEK, and PETG fall between these types. They show stiffness gains with higher RD and stress softening or buckling/slipping at high RH.

**Figure 10:**
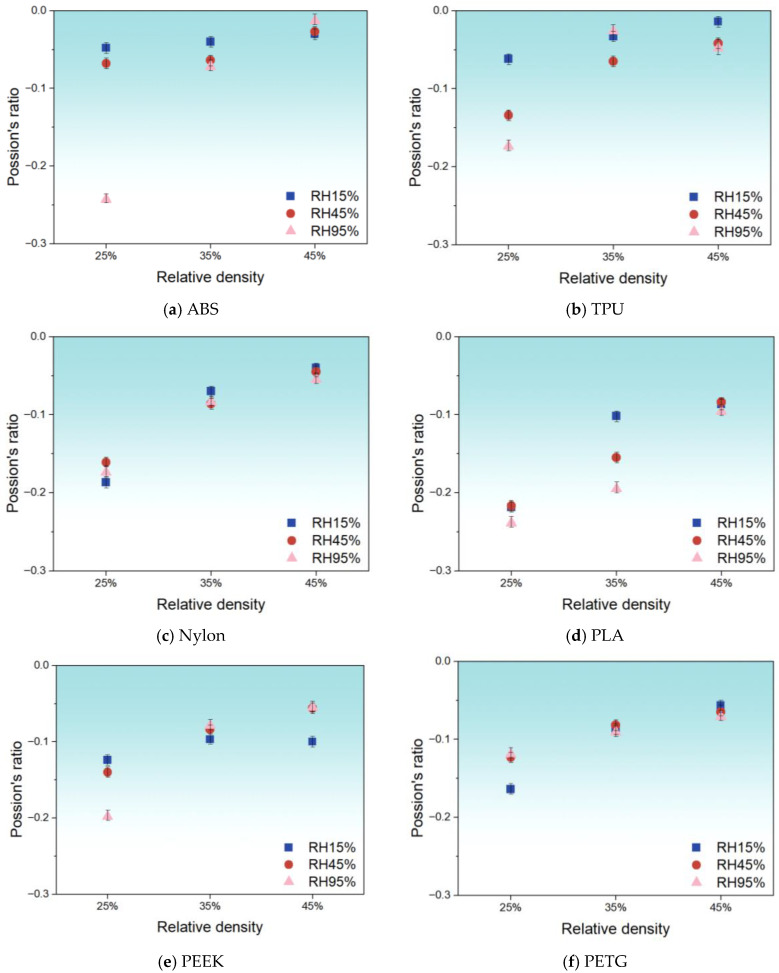
The evolution of Poisson’s ratio with increasing RD (25%, 35%, and 45%) under compressive loading at 15%, 45%, and 95% RH.

Figure 10 further illustrates how Poisson’s ratio varies according to RD under different RH conditions for each metamaterial. All metamaterials exhibit a negative Poisson’s ratio. For ABS, Poisson’s ratio increases with RD at all RH levels. The 95% RH samples show a marked change in Poisson’s ratio as structure changes. For TPU, Poisson’s ratio increases slowly with RD at 15% and 45% RH. At 95% RH, the lowest Poisson’s ratio occurs at 25% RD. It peaks at 35% RD and then decreases again at 45% RD. For Nylon, Poisson’s ratio remains nearly constant across all RD and RH levels. For PLA, trends differ according to the RH. At 15% RH, the increase in Poisson’s ratio slows with higher RD. At 45% RH, Poisson’s ratio rises uniformly. At 95% RH, the increase accelerates. At 25% and 45% RD, Poisson’s ratios are similar across RH. A significant difference appears at 35% RD: the highest Poisson’s ratio at 15% RH and the lowest at 95% RH. For PEEK, Poisson’s ratios at 35% RD are similar for all RH levels. At 25% and 45% RD, clear differences emerge. At 25% RD, the lowest Poisson’s ratio occurs at 95% RH. At 45% RD, it is lowest at 15% RH. For PETG, Poisson’s ratio trends with RD are similar across RH levels, except that at 15% RH and 25% RD, the Poisson’s ratio is minimal. Additional testing was also performed on 3D-printed wood samples under both low- and high-humidity conditions at various RDs. The results show that the Poisson’s ratio increases gradually with higher RD across low and high RH levels, while decreasing with increasing RH at the same RD. Detailed results can be found in Appendix A.3.

Overall, materials’ Poisson’s ratio responses exhibit distinct humidity-dependent regimes. Under low-to-moderate RH levels, most materials (ABS, TPU, PETG, and wood) demonstrate relatively stable gradual increases in Poisson’s ratio with rising RDs, while PLA shows diminishing growth rates in this regime. Notably, Nylon maintains exceptional stability across all humidity conditions. Upon entering high-humidity conditions, material behaviors undergo significant transformations: ABS displays anomalous deviations, TPU develops non-monotonic fluctuations (initial rise followed by decline), PLA exhibits accelerated responses, and PEEK manifests inversed humidity dependence at different RDs. Although PETG is less affected by high humidity, it still experiences minimum-value shifts. Collectively, material responses remain predictable at low-to-moderate humidity, whereas high humidity serves as a critical threshold inducing abrupt Poisson’s ratio behavioral shifts.

The variation in negative Poisson’s ratio further reflects the coupling between RH and structural geometry. A negative Poisson’s ratio mainly arises from unit cell hinge or strut rotation mechanisms. As the RD increases, the hinge count or strut cross-section grows. The overall deformation becomes more linear. The magnitude of the negative Poisson’s ratio decreases. Hence, Poisson’s ratio for most materials rises (moves closer to zero) with a higher RD. When the RH increases, water-induced plasticization alters local hinge compliance. In highly hydrophilic materials, plasticization eases hinge rotation and thus amplifies the negative Poisson’s ratio. In hydrophobic materials, plasticization does not significantly change hinge stiffness, so Poisson’s ratio remains nearly constant across RH levels. This insight into micro–macro coupling mechanisms offers theoretical and engineering guidance for material selection and metamaterial design in humidity-controlled environments.

## 4. Conclusions

This study systematically evaluated the moisture-adsorption behavior and mechanical performance of several common FFF polymers (ABS, TPU, Nylon, PLA, PEEK, and PETG) under controlled RH (15%, 45%, and 95%) and RD (25%, 35%, and 45%) conditions. By integrating mass-gain kinetics with tensile and compressive testing—alongside measurements of Young’s modulus, maximum strain, ultimate strength, and Poisson’s ratio—the coupled effects of water uptake, capillary condensation, and/or matrix absorption leading to effective pore occlusion, and architecture on metamaterial response, have been elucidated. Key findings are the following:

(1) All materials exhibit an initial rapid absorption phase (0–12 h), followed by a plateau with minor fluctuations (12–60 h), and a late-stage rebound (60–100 h). Final mass-gain ranking: Nylon > PETG > PLA ≈ ABS > TPU ≈ PEEK.
(2) Nylon and TPU reach peak ductility and strength at 45% RH under tension, driven by water-induced capillary condensation and/or matrix absorption, leading to effective pore occlusion and enhanced interlayer slip. Meanwhile, PEEK and ABS are reliable for moisture-sensitive applications.
(3) Mechanical metamaterials show the lowest peak stress at 25% RD under compression. Increasing RD raises peak stress across the board. For ABS, TPU, and PETG, the mechanical behavior at 35% and 45% RD is similar. In contrast, Nylon, PLA, and PEEK exhibit a stronger dependence of mechanical performance on RD—especially PLA, where the differences are most pronounced.
(4) All mechanical metamaterials maintain a negative Poisson’s ratio but differ in RD–RH sensitivity: ABS increases with RD at all RH values; TPU and PLA show RD trends that shift with humidity; Nylon is invariant; and PEEK and PETG are stable at 35% RD but exhibit RH-dependent minima at 25% and 45% RD. Collectively, material responses remain predictable at low-to-moderate humidity, whereas high humidity serves as a critical threshold inducing abrupt Poisson’s ratio behavioral shifts.

For humid-environment application, moderate humidity conditioning (≈45% RH) can be harnessed to boost flexibility in hygroscopic materials (Nylon and TPU), whereas low-uptake polymers (PETG, ABS, and PLA) ensure consistent performance. It may be advisable to modify the metastructure of ABS and PLA to reduce their sensitivity to various RH values in environments. Adjusting the fill density enables a balance between stiffness and deformability and allows for the customization of lateral deformation (Poisson’s ratio) to meet specific load requirements. In the future, physiological fluids in the human body will be adopted to simulate environmental change to study their performance and extend to real biomedical applications.

## Figures and Tables

**Table 1:** Printing parameters of materials in this study.

Material	Nozzle Temp. (°C)	Bed Temp. (°C)	Speed (mm/s)	Layer Height (mm)	Filament Supplier
ABS	250	90	30	0.1	D-BSDF ABS
TPU	220	50	30	0.1	PolyFlex TPU95 (Polymaker, Houten, Netherlands)
Nylon	280	50	30	0.1	PolyMide PA6-CF (Polymaker, Houten, Netherlands)
PLA	215	60	30	0.1	Prusa Galaxy Black PLA (Prusa Research, Prague, Czech Republic)
PEEK	400	120	15	0.1	Intamsys (INTAMSYS Technology GmbH, Eschborn, Germany)
PETG	240	80	30	0.1	PolyLite PETG (Polymaker, Houten, Netherlands)

**Figure A1:**
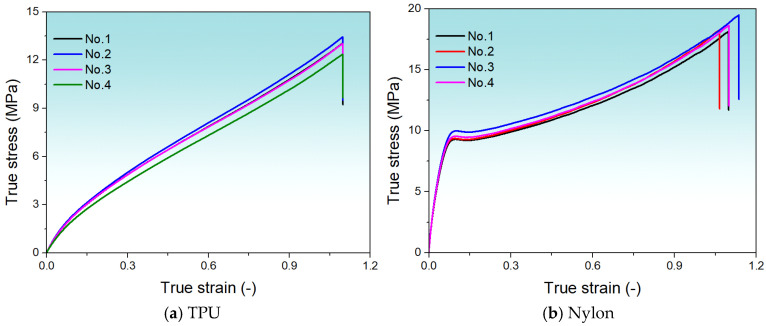
The repeated results of (**a**) TPU and (**b**) Nylon at RH 15%.

**Figure A2:**
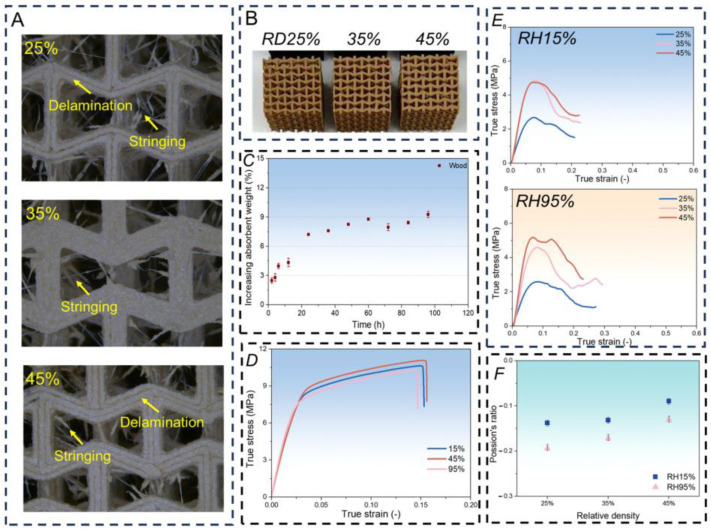
(**A**) Unit cell structure of the 3D-printed wood material with different RDs, and (**B**) its photographs. (**C**) Water absorption performance of 3D-printed wood. (**D**) Mechanical behavior under tensile loading at various humidity levels. (**E**) Compression performance of wood metamaterials at different RDs under varying RH values, and (**F**) the corresponding changes in Poisson’s ratio.

## Data Availability

The data presented in this study are available on request from the corresponding authors.

## Appendix A

### Appendix A.1. Repeated Results of TPU and Nylon at RH 15%

Figure A1 shows the four results of (a) TPU and (b) Nylon at RH 15%, respectively. It is clear that there is not much difference between each sample, thus indicating good reproducibility. The data are therefore convincing.

### Appendix A.2. Summarized Tensile Properties of All Materials

Table A1 shows the detailed values of Young’s modulus, maximum strain, and ultimate tensile strength (UTS) of each material at 15%, 45%, and 95% RH. The error bars denote the standard error (SE) of the mean mechanical properties.

**Table A1:** The Young’s modulus, maximum strain, and ultimate tensile strength (UTS) of each material at 15%, 45%, and 95% RH. The error bars denote the standard error (SE) of the mean mechanical properties.

	Young’s Modulus (MPa)			Maximum Strain (%)			UTS (MPa)		
RH%	15	45	95	15	45	95	15	45	95
ABS	371.518 ± 78.216	394.856 ± 64.229	382.020 ± 88.507	0.081 ± 0.011	0.069 ± 0.011	0.077 ± 0.014	12.404 ± 3.082	12.645 ± 2.589	12.587 ± 3.278
TPU	46.436 ± 10.563	42.443 ± 4.165	43.859 ± 8.372	2.000 ± 0.000	8.858 ± 0.595	10.044 ± 0.665	10.275 ± 2.013	23.437 ± 3.112	28.243 ± 7.120
Nylon	261.548 ± 22.601	175.002 ± 15.867	67.743 ± 0.781	1.477 ± 0.905	7.081 ± 0.679	7.247 ± 0.463	12.655 ± 3.234	18.017 ± 1.039	14.841 ± 1.031
PLA	406.939 ± 64.354	532.609 ± 79.684	462.855 ± 34.110	0.080 ± 0.028	0.077 ± 0.010	0.080 ± 0.028	11.040 ± 2.214	13.877 ± 0.932	12.086 ± 0.651
PEEK	563.447 ± 47.219	576.983 ± 48.710	547.799 ± 62.405	0.050 ± 0.117	0.045 ± 0.006	0.059 ± 0.028	11.994 ± 1.464	14.038 ± 0.909	12.621 ± 1.061
PETG	344.953 ± 41.337	355.370 ± 55.546	379.055 ± 73.427	0.075 ± 0.005	0.089 ± 0.016	0.086 ± 0.020	14.161 ± 2.269	14.206 ± 2.916	14.240 ± 2.677
Wood	345.822 ± 39.825	344.635 ± 50.400	379.309 ± 87.817	0.149 ± 0.026	0.135 ± 0.029	0.158 ± 0.038	8.297 ± 1.293	8.155 ± 1.400	8.345 ± 1.496

### Appendix A.3. Detailed Results of 3D-Printed Wood Samples

In addition to the six materials tested above, 3D-printed wood samples were evaluated using the same methods. The results are shown in Figure A2. Wood’s water absorption lies between the water absorption levels of the six materials. Tensile tests show no significant change in performance with humidity. Compression tests reveal more stringing at 25% RD and more pronounced delamination at 45% RD. Overall, the compression behavior of wood is strongly affected by both humidity and structure.
